# Risk of symptomatic osteoarthritis associated with exposure to ergonomic factors at work in a nationwide Italian survey

**DOI:** 10.1007/s00420-022-01912-1

**Published:** 2022-07-28

**Authors:** Angelo d’Errico, Dario Fontana, Gabriella Sebastiani, Chiara Ardito

**Affiliations:** 1 Epidemiology Unit ASL TO3, Grugliasco, TO Italy; 2 National Institute of Statistics (ISTAT), Rome, Italy; 3 Department of Economics and Statistics “Cognetti de Martiis”, University of Turin, Lungo Dora Siena 100A, 10153 Turin, Italy; 4 LABORatorio R. Revelli—Centre for Employment Studies, Turin, Italy; 5 NETSPAR—Network for Studies on Pensions, Aging and Retirement, Tilburg, The Netherlands

**Keywords:** Job-exposure matrix, Occupational health, Osteoarthritis, Musculoskeletal disorders, Ergonomic factors, Physical factors

## Abstract

**Objective:**

The risk of developing osteoarthritis (OA) has been reported to increase with exposure to various ergonomic factors at work, although this finding is still debated in the literature. Aim of this study was to assess the association between prevalence of symptomatic OA and exposure to workplace ergonomic factors assigned through a job-exposures matrix (JEM).

**Methods:**

The study population was composed of 24,604 persons of 40–69 years who participated in the National Health Survey 2013 and were employed at that occasion. Exposure to ergonomic factors was assigned to the study population through a JEM constructed from the Italian O*NET database, consisting of 17 physical factors, which were summed and averaged by job title (796 jobs) to obtain a combined exposure index. The outcome was self-reported OA characterized by moderate or severe limitations in daily activities. The relationship between OA prevalence and the combined exposure index in quartiles was examined using robust Poisson regression models adjusted for socio-demographics and potential confounders.

**Results:**

In the analysis adjusted for age and gender, the risk of OA was increased by approximately 20–30% in the second and third quartiles, and by 80% in the highest exposure quartile, compared to the least exposed, with a risk attenuation by approximately 15–20% controlling for other significant covariates.

**Conclusion:**

Our results support a causal role of exposure to physical factors at work in the development of OA. As OA is associated with a great burden of disability, any effort should be made to reduce workers’ exposure to ergonomic factors.

## Introduction

Osteoarthritis (OA) is a disorder characterized by degeneration of the articular cartilage, although it involves the whole joint, including synovium, ligaments, and subchondral bone (Buckwalter and Martin 2006). OA may cause joints pain, stiffness, swelling and physical limitation, but there is generally low correspondence between radiographic signs of OA and symptoms, with absence of symptoms in up to 50% of subjects with OA according to radiographic criteria (Hannan et al. 2000). The joints more commonly affected are knee, hip, hand, feet and spine, with high proportions of subjects reporting symptoms in more than one joint (Badley et al. 2021).

Symptomatic OA is common, with a prevalence around 20% in the adult population, which increases sharply with age, in particular over 45–50 years, with about one third of subjects over 65 years affected (Murphy et al. 2017). An incidence of OA in any body region of approximately 9 cases per 1,000 people older than 15 years has been estimated in UK for 2010, with a substantial increase in incidence between 2003 and 2010 also among younger subjects (Yu et al. 2015).

Regarding specific body regions, a prevalence of 3.6% has been estimated worldwide for symptomatic knee OA in both genders, higher in females (4.7%) than in males (2.6%) (Vos et al. 2012). In contrast, prevalences of 10–25% have been reported for symptomatic knee OA and hand OA among aged subjects, with a lower prevalence for symptomatic hip OA (Lawrence et al. 2008). OA is a cause of substantial disability, with knee and hip OA alone causing 2.2% of years lived with disability for all causes (Vos et al. 2012).

Several systemic factors have been found associated with the development of OA, including genetic factors (Valdes and Spector 2011), injuries (Wilder et al. 2002), deficiency of vitamin D, C and K (O’Neill et al. 2018), gender (Tschon et al. 2021), sex hormones and parity (Hellevik et al., 2017; Liu et al. 2009), diabetes (Dawson et al. 2018), hypertension (Zhang et al. 2017) and cardiovascular diseases (Wang et al. 2016). Based on current knowledge, OA appears mainly caused by mechanical overloading of the joints in performing physical activity at work or in the leisure time, by joint injuries, and by overweight/obesity (Richmond et al. 2013). However, high body mass index (BMI) would act also through systemic factors, as suggested by an increased risk of OA associated with overweight also in the hand, a region not under mechanical load from body weight (Jiang et al. 2016). Joint injury and malalignment would also concur to alter the distribution of loads in the joint, causing an increased pressure on selected parts of the joint, which in the long term leads to inflammatory processes, cartilage degeneration and changes in the joint structure characteristic of OA (Buckwalter and Martin 2006). Regarding physical activity (PA), while moderate PA has not been found associated with OA, exposure to heavy physical activity in leisure time has been reported to increase substantially the risk of knee (McAlindon et al. 1999) and hip OA (Vingård et al. 1993, 1998). Higher risks of knee OA have also been found in some categories of professional athletes, such runners, soccer players and weightlifters (Kujala et al. 1995), as well as of elbow OA in baseball pitchers (Buckwalter and Lane 1997).

Exposure to several ergonomic factors at work has been reported to increase the risk of OA, including heavy physical workload, frequent bending, kneeling or standing, repetitive movements and vibration, although most of the available evidence concerns knee and hip OA (McWilliams et al. 2011; Sulsky et al. 2012; Yucesoy et al. 2015; Verbeek et al. 2017; Gignac et al. 2019; Sun et al. 2019; Wang et al. 2020). A recent meta-analysis from the WHO/ILO programme on the work-related burden of disease estimated a more than two-fold risk of knee or hip OA associated with exposure to physical factors at work for more than two hours per day, including force exertion, awkward postures, repetitiveness, hand-arm vibration, lifting, kneeling and/or squatting, and climbing (Hulshof et al. 2021).

However, most studies included in these reviews evaluated the association of occupational exposure to ergonomic factors with knee or hip OA, whereas are less common those focussing on OA in other joints or in any body region. For hand OA, several occupational factors have also been found to increase the risk, including heavy lifting, repetitive movements, working at a fast pace, few rest breaks (Rossignol et al. 2005; Fontana et al. 2007). For elbow OA, a case–control study found strong associations with self-reported exposure to force, repetitive movements, and vibration in the previous ten years (Spahn et al. 2017).

In general, the available evidence appears limited by the fact that many studies in this field are potentially affected by methodological problems, such as differential exposure misclassification, selection, and uncontrolled confounding by sociodemographic, behavioural, and biological risk factors (Hulshof et al. 2021). In particular, differential exposure misclassification appears a major threat to the validity of the associations observed between physical factors and OA, due to the fact that the great majority of studies assessed it through self-report within cross-sectional or case–control studies.

The present study aimed at examining the risk of OA in any joint associated with exposure to ergonomic factors at work assessed through a Job-Exposure Matrix (JEM), in a large Italian survey. As exposure assessment through JEMs is independent from individuals’ self-reported health status, their application in epidemiological studies prevents differential misclassification bias of the exposure and provides unbiased estimates of the association between exposure and outcome also in cross-sectional and retrospective studies (Peters 2020).

## Materials and methods

### Data collection

#### Study population

Data from the 2013 National Health Survey (NHS), conducted by the Italian National Institute of Statistics (hereafter: ISTAT) on a representative sample of the Italian population, were used to get information on sociodemographic factors, lifestyles, biological CVD risk factors, job title, symptomatic OA and comorbidities. In Italy, this survey is carried out periodically, generally every 5 years (Odone et al. 2018). The survey collects detailed information on individual and household socioeconomic characteristics and on health conditions, including perceived health, long-term chronic diseases, and functional limitations, as well as on lifestyles and use of health services. The survey is based on a two-stage sampling, with municipalities as primary sampling units and households as secondary sampling units. For each household, information is gathered for all the members belonging to the family unit, partly through face-to-face interviews and partly through a self-administered questionnaire, the latter mainly used to collect information on health conditions and lifestyles. For the 2013 survey, ISTAT collected information on 119,073 subjects from 1,456 municipalities, with a participation rate of 82.5% (Fabiani et al. 2016).

**Fig. 1:**
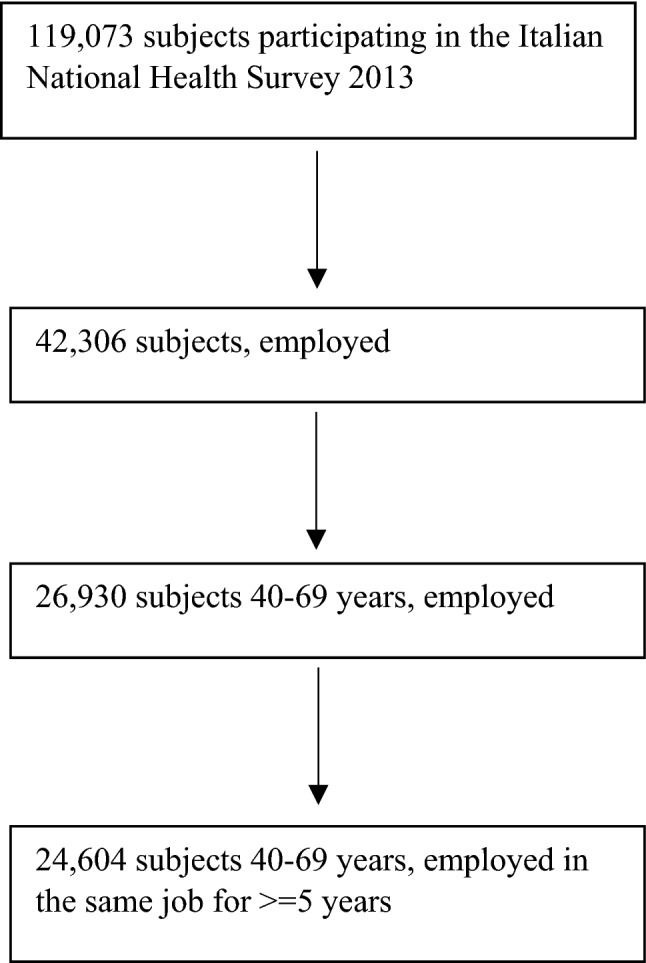
Selection of the study population

The study was restricted to employed men and women, because information on job codes with the maximum level of detail (ISTAT’s Classification of Professions, CP 2011 5-digit), which was needed to link exposure scores in the JEM to survey data, was available only for subjects still in employment. The study population was further restricted to subjects 40–69 years, given the very low prevalence of symptomatic OA among younger people, as well as to workers who reported to have worked in the same job for at least five years, to guarantee a minimum exposure duration (Fig. 1). The final sample included 14,812 men and 9,792 women.

#### Exposure assessment

Exposure to ergonomic factors was assigned to the study population through a JEM constructed from the Italian O*NET database. O*NET contains information on hundreds of physical and mental descriptors, in terms of skills, knowledge, activities, work context, etc., aggregated at the job level (www.onetcenter.org). The Italian O*NET database includes scores of all these dimensions, constructed from workers’ self-reports, based on interviews of approximately 20 workers for each of the 796 jobs of the Italian classification (CP2011, 5-digit level). For each job, the O*NET database contains scores for each descriptor, rated by importance, frequency, or level of a certain workplace characteristic. Answers to these questions are collected on 5-point or 7-point level, depending on the item, which represent the score assigned by each worker to a certain work characteristic, averaged for each of the 796 occupations.

From the hundred variables available in O*NET, a JEM was constructed on 21 physical factors, which were further reduced through Principal Component Analysis to 17 factors potentially associated with musculoskeletal disorders. For all 17 factors, good reliability against the same items of a corresponding US O*NET JEM has been shown (d’Errico et al. 2019) (Table 1). Of the 17 items, 3 focussed on force exertion (static strength, dynamic strength, trunk strength), 6 on activity level and repetitive movements of the upper limb (manual dexterity, fingers dexterity, wrist-finger speed, handling and moving objects, time spent making repetitive motions, time spent using hands to handle, control, or feel objects, tools or controls), 4 on postures (awkward positions; standing; kneeling, crouching, stooping, or crawling; bending and twisting the body), 2 items on activities involving the whole body (performing generalized physical activity; walking and running), 2 items on exposure to vibration (whole-body vibration, driving vehicles or other types of moving machinery).

**Table 1:** Description of the items in the Italian O*NET databases used to construct the composite ergonomic index

Exposure	Domain	Question/definition	Response scale
*Force exertion*			
Dynamic Strength	Abilities	The ability to exert muscle force repeatedly or continuously over time. This involves muscular endurance and resistance to muscle fatigue	Importance: 1–5 Level: 1–7
Static Strength	Abilities	The ability to exert maximum muscle force to lift, push, pull, or carry objects	Importance: 1–5 Level: 1–7
Trunk Strength	Abilities	The ability to use your abdominal and lower back muscles to support part of the body repeatedly or continuously over time without 'giving out' or fatiguing	Importance: 1–5 Level: 1–7
*Activity level and repetitive movements of the upper limb*			
Wrist-Finger Speed	Abilities	The ability to make fast, simple, repeated movements of the fingers, hands, and wrists	Importance: 1–5 Level: 1–7
Finger Dexterity	Abilities	The ability to make precisely coordinated movements of the fingers of one or both hands to grasp, manipulate, or assemble very small objects	Importance: 1–5 Level: 1–7
Manual Dexterity	Abilities	The ability to quickly move your hand, your hand together with your arm, or your two hands to grasp, manipulate, or assemble objects	Importance: 1–5 Level: 1–7
Handling and Moving Objects	Activities	Using hands and arms in handling, installing, positioning, and moving materials, and manipulating things	Importance: 1–5 Level: 1–7
Time Making Repetitive Motions	Context	How much does this job require making repetitive motions?	Frequency: 1–5
Time Using Your Hands	Context	How much does this job require using your hands to handle, control, or feel objects, tools or controls?	Frequency: 1–5
*Postures*			
Awkward Positions	Context	How often does this job require working in cramped work spaces that requires getting into awkward positions?	Frequency: 1–5
Time Standing	Context	How much does this job require standing?	Frequency: 1–5
Time Kneeling, Crouching, Stooping, or Crawling	Context	How much does this job require kneeling, crouching, stooping or crawling?	Frequency: 1–5
Time Bending or Twisting the Body	Context	How much does this job require bending or twisting your body?	Frequency: 1–5
*Vibration*			
Exposed to Whole Body Vibration	Context	How often does this job require exposure to whole-body vibration (e.g., operate a jackhammer)?	Frequency: 1–5
Driving Vehicles, Mechanized Devices, or Equipment	Activities	Running, manoeuvring, navigating, or driving vehicles or mechanized equipment, such as forklifts, passenger vehicles, aircraft, or watercraft	Importance: 1–5 Level: 1–7
*Whole-body activities*			
General Physical Activities	Activities	Performing physical activities that require considerable use of your arms and legs and moving your whole body, such as climbing, lifting, balancing, walking, stooping, and handling of materials	Importance: 1–5 Level: 1–7
Spend Time Walking and Running	Context	How much does this job require walking and running?	Frequency: 1–5

Importance ranges from 1 (lowest) to 5 (highest); Level ranges from 1 (lowest) to 7 (highest); Frequency ranges from 1 (never) to 5 (all the time)

Scores of each item were standardized on a 0–100 scale and averaged, to compute a composite ergonomic exposure index (Cronbach alpha = 0.90). Among the factors selected, those belonging to the “ability” and the “activity” domains are scored for both importance of a certain characteristic in a job and for the level of the characteristic, such as the level of an ability needed to perform a job or the level of an activity typical of that job. For these factors (manual and finger dexterity, trunk strength, handling and moving objects), importance is scored from 1 to 5, whereas level ranges from 1 to 7 (Table 1). In contrast, the other 7 factors, which belong to the “context” domain and focus on aspects of both job content and on workplace characteristics, are collected on a frequency scale from 1 to 5 (from never to all the time, or every day). ‘Level’ scores of items in the ‘work ability’ and ‘work activities’ domains were reclassified to a level equal to zero, if their importance score was below or equal to 1.

In the study population, the composite ergonomic exposure index (Ergo-index) had a mean equal to 25.6 (s.d. 14.3) and a range of scores of 2.9–60.8. The Ergo-index was strongly correlated with most items composing it, with all correlations above 0.70, except for whole-body vibration, driving and awkward postures (Table 2). For the analysis, it was categorized in four ordinal groups, with cut-offs in correspondence of the median (27.4) and the interquartile distribution (15.94, 35.68) of the original JEM.

**Table 2:** Pearson’s correlation coefficients between the Ergo-Index and each exposure item composing it

Exposure	Pearson’s *r*
Handling and moving objects	0.93
Bending and twisting the body	0.88
Finger dexterity	0.79
Manual dexterity	0.90
Use of hands to handle and control	0.86
Time spent making repetitive motions	0.73
Kneeling, crouching, stooping or crawling	0.80
Awkward postures	0.65
Walking and running	0.74
Standing	0.81
Trunk strength	0.88
Static strength	0.89
Dynamic strength	0.86
Whole-body vibration	0.49
Generalized physical activity	0.88
Driving	0.60
Wrist-finger speed	0.77

#### Outcome

The outcome of the study was: “Self-reported doctor-diagnosed OA, for which drugs were taken in the previous 12 months, combined with moderate or severe limitations in daily activities”, defined as “symptomatic OA”. In detail, subjects were asked if they were affected by OA in any body region, if the disease was diagnosed by a physician, and whether they took any drug for the disease during the previous 12 months. The presence of physical limitations was ascertained through a question on limitations in normal daily activities lasting at least six months, with possible answers: severe limitations, non-severe limitations, no limitations.

#### Covariates

Information on potential confounding factors was collected through questionnaires in the NHS survey, including socio-demographics (age, gender, geographical area of residence), engagement in domestic work, leisure physical activity, smoking habit, overweight/obesity, diabetes, hypertension, cardiovascular diseases (CVD).

Overweight and obesity were derived from the body mass index (BMI), calculated on the self-reported height and weight in the survey, according to a standard procedure. Based on the WHO classification, BMI was categorized in normal weight (18.5 <  = BMI < 25), underweight (BMI < 18.5), overweight (25 <  = BMI <  = 30), and obese (BMI > 30). Information on diabetes, hypertension, and CVD at the time of the interview was collected through yes/no questions. Smoking habit was classified into five categories of lifetime smoking history, according to pack-years (py) smoked: never smoker (0 py), 0.1–10 py, 10.1–20 py, 20.1–30 py, and > 30 py.

### Data analysis

The frequency distribution of covariates between workers affected or not by symptomatic OA was compared using chi-square statistics, for categorical variables, and *t* test, for continuous ones.

The relationship between prevalence of symptomatic OA and exposure to physical factors, represented by the Ergo-index kept continuous or categorized in quartiles, was examined by means of Poisson regression models with the Huber-White sandwich estimator of variance, also known as Poisson robust regression models. These models were used to avoid overestimation of the variance, which is known to affect confidence intervals of relative risks in Poisson models when applied to binomial data (Barros and Hirakata 2003).

A first analysis was adjusted only for age (5-year age classes), gender, and geographical area of residence (4 areas). In a second model, BMI (normal weight, overweight, obese, underweight), pack-years of smoking (5 categories), number of hours of domestic work per week (continuous), leisure physical activity (intense or regular, light, no activity), diabetes (yes/no), hypertension (yes/no) and prevalent CVD (yes/no) (including coronary heart disease and stroke) were also included as adjustment variables. The association between covariates and OA risk was assessed through the analysis based on Ergo-index quartiles. No adjustment was performed for educational level, as it was too strongly correlated with the Ergo-index (Spearman rho = 0.52), so there was concern about multicollinearity in the regression model (Vatcheva et al. 2016). The high correlation between education and exposure to ergonomic factors was partly caused by the use of a JEM, as education is known to strongly influence selection into job type (e.g., heavy manual, light manual, non-manual occupations). Furthermore, because JEMs provide overall mean scores by occupation, with no exposure variability within occupations, this may artificially increase the correlation between educational attainment and exposure to work factors displaying a social gradient.

Analyses were not stratified by gender, as in preliminary analyses adjusted for age and geographical area no significant interaction was found between gender and Ergo-index quartiles (all *p* values > 0.28).

## Results

### Descriptive analyses

In Table 3 are presented descriptive statistics of covariates used in the analysis by presence/absence of symptomatic OA. Overall, 907 workers (3.7%) reported to be affected by symptomatic OA, with a higher prevalence among women, as well as in subjects overweight/obese, resident in the North-East or in the South of Italy, affected by hypertension, diabetes or CVD, not physical active in leisure time, and engaged in more hours of domestic work. For age and smoking history, significant trends of increasing OA prevalence with increasing age and number of pack-years smoked were also noted.

**Table 3:** Sociodemographic characteristics, smoking history, BMI, domestic work hours, and prevalence of diabetes, hypertension and cardiovascular diseases (CVD), by presence/absence of Symptomatic Osteoarthritis (OA)

Covariates	Without symptomatic OA		With symptomatic OA		*P* value
	*N*	%	*N*	%	
Gender					
Male	14,415	97.3	397	2.7	< 0.001
Female	9282	94.8	510	5.2	
Age					
40–44	5795	98.7	74	1.3	< 0.001
45–49	6323	97.9	136	2.1	
50–54	5409	95.5	252	4.5	
55–59	4123	93.7	279	6.3	
60–64	1636	92.0	143	8.0	
65–69	411	94.7	23	5.3	
Geographical area					
North-West	5805	97.1	171	2.9	< 0.001
North-East	5415	96.2	212	3.8	
Centre	4577	96.9	157	3.3	
South	7900	95.6	367	4.4	
Pack-years (py)					
Never smoker (0 py)	11,372	97.3	320	2.7	< 0.001
0.1–10 py	4209	95.9	178	4.1	
10.1–20 py	3205	96.0	132	4.0	
20.1–30 py	2323	95.3	115	4.7	
> 30 py	2424	93.9	157	6.1	
Missing	164	97.0	5	3.0	
Body mass index (BMI)					
Underweight	371	97.1	11	2.9	< 0.001
Normal weight	11,721	97.1	350	2.9	
Overweight	9060	96.0	378	4.0	
Obese	2545	93.8	168	6.2	
Hypertension					
Yes	4249	92.6	341	7.4	< 0.001
No	19,448	97.2	566	2.8	
Diabetes					
Yes	812	91.4	76	8.6	< 0.001
No	22,885	96.5	831	3.5	
Cardiovascular diseases					
Yes	396	88.0	54	12.0	< 0.001
No	23,301	96.5	853	3.5	
Leisure physical activity					
Intense or regular	7019	97.5	179	2.5	< 0.001
Light	6157	95.6	280	4.4	
No activity	10,521	95.9	448	4.1	
Occupational class					
Employers, professionals, executives	3129	13.2	61	6.7	
Administrative workers and technicians	9105	38.4	339	37.4	
Artisans, traders	3999	16.9	155	17.1	< 0.001
Skilled and unskilled workers	7187	30.3	339	37.4	
Missing	277	1.2	13	1.4	

	Mean	St. dev	Mean	St. dev	*P* value
N. hours of domestic work per week	11.7	12.0	16.1	14.4	< 0.001

### Multivariate analyses

Table 4 displays the Prevalence Ratios (PRs) estimated through Poisson robust regression models adjusted for age, gender, and area of residence group (left), and fully adjusted (right).

**Table 4:** Prevalence Ratios (PR) of symptomatic Osteoarthritis (OA) for exposure to ergonomic factors (quartiles of the ergonomic score), sociodemographic characteristics, smoking history, diabetes, hypertension, cardiovascular diseases—poisson robust regression models

Exposure	Model 1 PR	95% CI	Model 2 PR	95% CI
Ergonomic score (ref.: First quartile)	1	–	1	–
Second quartile	1.31	1.07–1.61	1.21	0.99–1.49
Third quartile	1.23	1.03–1.47	1.14	0.96–1.37
Fourth quartile	1.80	1.53–2.11	1.63	1.38–1.92
Area of residence (ref.: North-West)	1	–	1	–
North–East	1.28	1.05–1.56	1.24	1.02–1.51
Centre	1.12	0.91–1.39	1.08	0.87–1.34
South	1.53	1.28–1.82	1.41	1.18–1.69
Gender (ref.: Males)	1	–	1	–
Females	2.24	1.97–2.55	2.38	2.00–2.84
Age class (ref: 40–44 years)	1		1	–
45–49 years	1.68	1.26–2.22	1.61	1.21–2.13
50–54 years	3.56	2.75–4.61	2.99	2.30–3.90
55–59 years	5.11	3.96–6.60	3.78	2.90–4.93
60–64 years	6.68	5.07–8.82	4.65	3.48–6.22
65–69 years	4.91	3.11–7.74	3.29	2.06–5.24
Smoking history (ref: Never smokers)			1	–
0.1–10 pack-years			1.56	1.30–1.87
10.1–20 pack-years			1.54	1.26–1.88
20.1–30 pack-years			1.79	1.45–2.21
> 30 pack-years			1.77	1.45–2.15
BMI category (ref: Normal weight)			1	–
Overweight			1.32	1.14–1.53
Obese			1.66	1.37–2.00
Underweight			0.89	0.50–1.59
Leisure physical activity (ref: intense/regular)			1	–
Light			1.21	1.00–1.46
No activity			1.17	0.98–1.39
Diabetes (ref.: No)			1	–
Yes			1.41	1.12–1.79
Hypertension (ref: No)			1	–
Yes			1.62	1.41–1.87
Cardiovascular diseases (ref: No)			1	–
Yes			1.93	1.48–2.51
Domestic work hours (continuous)				
1-h increase			1.01	1.00–1.02

Model 1 adjusted for age, gender and area of residence group. Model 2 adjusted for age, gender, area of residence group, BMI, pack-years of smoking, weekly hours of domestic work, leisure physical activity, diabetes, hypertension and prevalent CVD

In the analysis adjusted for age, gender and area of residence, the ergonomic score (Ergo-index) kept continuous was significantly associated with symptomatic OA, with an increase in the relative risk of OA equal to 1.015 (96% CI 1.011–1.020) for an increase of one point in the index score (results not shown). When the Ergo-index was categorized in quartiles, the risk of OA was increased by approximately 20–30% in the second and third quartiles (RR = 1.31, 95% CI 1.07–1.61 and RR = 1.23, 95% CI 1.03–1.47, respectively), while an increase in risk by 80% was estimated for the highest exposure quartile (RR = 1.80, 95% CI 1.53–2.11), compared to the least exposed (Table 3).

Controlling for the other covariates, the risk of OA associated with higher ergonomic exposure was attenuated by approximately 15–20% but remained statistically significantly associated both when the Ergo-index was treated as a continuous variable (RR = 1.013, 95% CI 1.008–1.020 for an increase of one point in the score), as well as categorizing it in quartiles.

No significant interaction was found between the ergonomic score quartiles and age on the OA risk, neither when age was divided in 5-year categories, or in only two classes (40–54 and 55–69 years), to preserve statistical power.

Among covariates, gender (RR = 2.24, 95% CI 1.97–2.55), overweight (RR = 1.32, 95% CI 1.14–1.53) and obesity (RR = 1.66, 95% CI 1.37–2.00), diabetes (RR = 1.41, 95% CI 1.12–1.79), hypertension (RR = 1.62, 95% CI 1.41–1.87), cardiovascular diseases (RR = 1.93, 95% CI 1.48–2.51), domestic work hours (RR = 1.010, 95% CI 1.005–1.016, for an increase in one hour per week), and pack-years of smoking (RR = 1.77, 95% CI 1.45–2.15, for the highest category) were independently associated with a higher OA risk. Living in South Italy also showed a marginally significantly higher OA risk, compared to living in North-West or Centre.

## Discussion

In this cross-sectional study, symptomatic OA was associated with exposure to ergonomic factors at work, assessed by means of a JEM, with a risk increased by about 80% in the highest vs. the lowest exposure quartile and a significant dose–response effect. This association was robust to the adjustment for several potential confounders, with only a slight attenuation in the fully adjusted model. The study is one of the few which examined, in the general employed population, the association of OA in any body region with exposure to physical factors at work assessed through a JEM, and not using self-reported working conditions.

Our results support the findings of several systematic reviews, which concluded that OA is associated with occupational exposure to physical factors, especially lifting and carrying heavy loads, kneeling, and squatting, and climbing (Mc Williams et al. 2011; Verbeek et al. 2017; Canetti et al. 2020; Wang et al. 2020). However, the great majority of the available studies were case–control or cross-sectional studies in which exposure assessment relied on self-reports, with the possibility that the observed associations were caused by recall bias, leading to differential exposure misclassification. Only a few studies used objective methods for exposure assessment, such as job-exposure matrices, expert ratings, or observations (Dembe et al. 2004; D’Souza et al. 2008; Jensen 2005; Martin et al. 2013; Rijs et al. 2014; Rubak et al. 2014), with some inconsistencies. Among these, knee OA was found associated with kneeling in three studies (Jensen 2005; D’Souza et al. 2008; Martin et al. 2013) and with lifting in one study (D’Souza et al. 2008). However, no significant association was observed by Rijs et al. (2014) between hip or knee OA and exposure to high force, uncomfortable postures, or repetitive movements in the current job, while Rubak et al. (2014) found only a modest association between hip replacement and cumulative exposure to lifting, limited to men. Using panel data, Dembe et al. (2014) found significant associations of self-reported doctor-diagnosed arthritis in any body region with several ergonomic factors, as well as with a composite ergonomic exposure index, assigned to the study population through a JEM applied to job histories. Consistency of our results with those of Dembe et al. (2014) is particularly relevant, as Dembe et al. (2014) employed methods very similar to ours, in terms of both exposure assessment, which was similarly performed through a JEM on physical exposures constructed from O*NET data, and outcome (self-reported doctor-diagnosed arthritis in any body region).

A recent meta-analysis by WHO/ILO on the work-related burden of hip and knee osteoarthritis associated with exposure to ergonomic factors concluded that the quality of evidence of a causal relationship is low and that the strength of evidence is limited, in spite a significantly increased meta-risk was computed (meta-RR = 2.20, 95% CI 1.42–3.40), with low heterogeneity of the estimates (Hulshof et al. 2021). Nonetheless, it is worth noting that the conclusions of this meta-analysis were based on only three case–control studies, two on knee OA (Seidler et al. 2008; Gholami et al. 2016) and one on hip OA (Yoshimura et al. 2000), as the strict inclusion criteria adopted excluded a large number of studies considered of low quality.

In the only prospective cohort study available on hip OA and occupational exposures, a two-fold risk of hip arthroplasty was estimated for exposure to intense physical activity at work (Flugsrud et al. 2002), suggesting that the associations reported in the literature with ergonomic factors are unlikely explained by biases related to study design. However, a cohort study on knee OA reported a high risk for exposure to very heavy physical work (OR = 18.3), although estimated on only 4 exposed cases, whereas the association was lower and not significant in the heavy physical work category (OR = 1.7, 95% CI 0.8–3.9) (Toivanen et al. 2010).

The consistency of our results on non-occupational risk factors for OA with findings reported in the literature seems to demonstrate the validity of the outcome used in the study. Significant associations were found in the fully adjusted model for most of the potential risk factors examined, including female gender (Tschon et al. 2021), overweight/obesity (Richmond et al. 2013), diabetes (Dawson et al. 2018), cardiovascular diseases (Wang et al. 2016), and hypertension (Zhang et al. 2017). Domestic physical activity was also associated with OA in one study (Kopec et al. 2017). In contrast, smoking has been found inversely associated with knee OA in a meta-analysis (Kong et al. 2017), whereas we observed a positive association with number of pack-years smoked. The slightly increased risk of OA associated with light physical activity and with inactivity appears the result of reverse causality, as pain or physical limitations, which were part of the outcome definition, would limit the possibility of performing physical activity.

### Strengths

A major strength of the present study is that it was based on a large representative national survey, allowing to examine with great statistical power the association of symptomatic OA with exposure to physical factors at work, as well as with non-occupational risk factors. As the survey was representative of the general population, the results of this study appear generalizable to the whole employed Italian population.

However, the main strength of the study appears the assignment of exposure to occupational factors through a JEM, as it protected the results from differential exposure misclassification, due to the possibility that individuals affected by OA may overestimate exposure to physical factors, which may lead to overestimation of the association with OA.

Furthermore, the availability in the survey of a large set of covariates allowed to adjust the estimates for several variables significantly associated with OA risk known as risk factors established from previous literature, which are the main potential confounders of the association between OA and ergonomic factors.

### Limitations

The use of a JEM protects from differential exposure misclassification but introduces a certain degree of non-differential exposure misclassification, because exposure scores are averaged for each job group without variability within occupations, with the likely consequence of a dilution of the association between ergonomic exposure at work and symptomatic OA.

The strong correlation observed between exposure to physical factors at work and educational level prevented from the inclusion of the latter variable in the regression models, to avoid multicollinearity, not identifiable using standard methods, such as Variance Inflation Factor (Vatcheva et al. 2016). Therefore, it cannot be excluded that the association between exposure to ergonomic factors and OA has been overestimated, due to residual confounding by educational level. For example, OA over-reporting among subjects with lower education could have biased the results away from the null.

Last, the cross-sectional design of the study does not allow to consider the association between higher exposure to ergonomic factors and OA prevalence as causal, although it is not plausible that reverse causation occurred, i.e., that workers affected by OA would be more likely to work in jobs with higher ergonomic exposure. Furthermore, because of the cross-sectional design, results were likely biased by selection, namely by healthy worker effect, as plausibly part of the workers affected by symptomatic OA moved, because of pain and physical limitations, into jobs less exposed to ergonomic factors in the years before the survey, with the consequence of an underestimation of the true association. However, the restriction operated to workers who stayed in same job during the previous five years could have limited the extent of such a selection bias.

## Conclusions

In this study, Italian workers employed in jobs with high exposure to ergonomic factors displayed a prevalence of symptomatic OA almost double than that of workers in jobs with low exposure, after considering the effect of several health and behavioural potential confounders. These results support a causal role of exposure to physical factors at work in the development of OA. Given the great burden of disability associated with OA, any effort should be made to reduce workers’ exposure to ergonomic factors.

## Data Availability

The data that support the findings of this study are available on request from the corresponding author. The data are not publicly available due to privacy or ethical restrictions.
